# Glycyrrhizin Production in Licorice Hairy Roots Based on Metabolic Redirection of Triterpenoid Biosynthetic Pathway by Genome Editing

**DOI:** 10.1093/pcp/pcad161

**Published:** 2023-12-28

**Authors:** Naoki Chiyo, Hikaru Seki, Takuya Kanamoto, Hiroshi Ueda, Mareshige Kojoma, Toshiya Muranaka

**Affiliations:** Department of Biotechnology, Graduate School of Engineering, Osaka University, 2-1 Yamadaoka, Suita, 565-0871 Japan; Department of Biotechnology, Graduate School of Engineering, Osaka University, 2-1 Yamadaoka, Suita, 565-0871 Japan; RIKEN Center for Sustainable Resource Science, 1-7-22 Suehiro-cho, Tsurumi-ku, Yokohama, 230-0045 Japan; Institution for Open and Transdisciplinary Research Initiatives, Osaka University, 2-1 Yamadaoka, Suita, 565-0871 Japan; Department of Biotechnology, Graduate School of Engineering, Osaka University, 2-1 Yamadaoka, Suita, 565-0871 Japan; Department of Biotechnology, Graduate School of Engineering, Osaka University, 2-1 Yamadaoka, Suita, 565-0871 Japan; School of Pharmaceutical Sciences, Health Sciences University of Hokkaido, Tobetsu-cho, Ishikari-gun, 061-0293 Japan; Department of Biotechnology, Graduate School of Engineering, Osaka University, 2-1 Yamadaoka, Suita, 565-0871 Japan; RIKEN Center for Sustainable Resource Science, 1-7-22 Suehiro-cho, Tsurumi-ku, Yokohama, 230-0045 Japan; Institution for Open and Transdisciplinary Research Initiatives, Osaka University, 2-1 Yamadaoka, Suita, 565-0871 Japan

**Keywords:** Genome editing, Glycyrrhizin, Hairy root culture, Metabolic engineering, Triterpenoids

## Abstract

Glycyrrhizin, a type of the triterpenoid saponin, is a major active ingredient contained in the roots of the medicinal plant licorice (*Glycyrrhiza uralensis, G. glabra* and *G. inflata*), and is used worldwide in diverse applications, such as herbal medicines and sweeteners. The growing demand for licorice threatens wild resources and therefore a sustainable method of supplying glycyrrhizin is required. With the goal of establishing an alternative glycyrrhizin supply method not dependent on wild plants, we attempted to produce glycyrrhizin using hairy root culture. We tried to promote glycyrrhizin production by blocking competing pathways using CRISPR/Cas9-based gene editing. *CYP93E3 CYP72A566* double-knockout (KO) and *CYP93E3 CYP72A566 CYP716A179 LUS1* quadruple-KO variants were generated, and a substantial amount of glycyrrhizin accumulation was confirmed in both types of hairy root. Furthermore, we evaluated the potential for promoting further glycyrrhizin production by simultaneous *CYP93E3 CYP72A566* double-KO and *CYP88D6*-overexpression. This strategy resulted in a 3-fold increase (∼1.4 mg/g) in glycyrrhizin accumulation in double-KO/*CYP88D6*-overexpression hairy roots, on average, compared with that of double-KO hairy roots. These findings demonstrate that the combination of blocking competing pathways and overexpression of the biosynthetic gene is important for enhancing glycyrrhizin production in *G. uralensis* hairy roots. Our findings provide the foundation for sustainable glycyrrhizin production using hairy root culture. Given the widespread use of genome editing technology in hairy roots, this combined with gene knockout and overexpression could be widely applied to the production of valuable substances contained in various plant roots.

## Introduction

Plant secondary metabolites are substances produced by plants to counteract external environmental stimuli and stresses such as herbivores, pathogens and ultraviolet light (Shitan 2016). Many plant secondary metabolites may have beneficial biological effects on human health and have been used in pharmaceuticals for a long time. Among them, triterpenoid saponins are a group of compounds consisting of multiple sugars attached to the carbon skeleton of triterpenes, and are known to be the major active ingredients in many herbal medicines (Zhao et al. 2010). Licorice (*Glycyrrhiza uralensis, G. glabra* and *G. inflata*), a member of the legume family and one of the most economically important medicinal plants (Hayashi and Sudo 2009), contains a triterpenoid saponin called glycyrrhizin in the root and stolon. Glycyrrhizin and its aglycone, glycyrrhetinic acid, have diverse pharmacological effects that may include anticancer, anti-inflammatory, hepatoprotective and antiviral properties (Ashfaq et al. 2011, Yang et al. 2014, Hsiang et al. 2015, Wang et al. 2016, Graebin 2018). Glycyrrhizin is also used worldwide as a natural sweetener in the confectionery and tobacco industries because it is 50–100 times sweeter than sugar (Hayashi and Sudo 2009, El-Lahot et al. 2017). The source of glycyrrhizin is wild or cultivated licorice; however, licorice roots take 3–4 years to grow from planting to harvest and growing demand has led to a recent decline in natural reserves of licorice due to overharvesting (Hayashi and Sudo 2009, Khaitov et al. 2022). Therefore, a sustainable licorice supply is required.

**Fig. 1:**
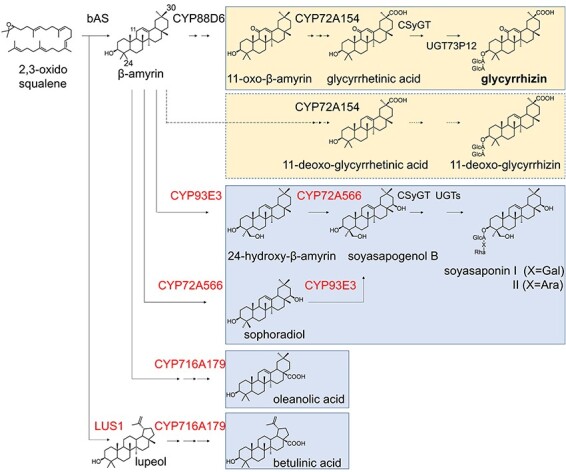
Proposed biosynthetic pathway of triterpenoids in licorice. Arrows indicate a cyclization, single oxidation or single glycosylation reaction. Dashed arrows indicate a single oxidation or single glycosylation reaction identified in double-KO and quadruple-KO hairy roots in this study. The target genes for knockout in this study are shown in red. Numbers on β-amyrin indicate carbon positions. Abbreviations: Ara, arabinose; bAS, β-amyrin synthase; Gal, galactose; GlcA, glucuronic acid; Rha, rhamnose; UGT, UDP-glycosyltransferase.

Licorice root contains various triterpenoids besides glycyrrhizin, such as soyasaponins, oleanolic acid and betulinic acid (Hayashi et al. 1988, 1990, 1993, Kojoma et al. 2010). These triterpenoids share a common precursor, 2,3-oxidosqualene, and glycyrrhizin, soyasaponins and oleanolic acid are derived from β-amyrin, a cyclized derivative of 2,3-oxidosqualene (**Fig. 1**). In the glycyrrhizin pathway, β-amyrin is oxidized at the C-11 position by the cytochrome P450 monooxygenase CYP88D6 to 11-oxo-β-amyrin (Seki et al. 2008), which is then oxidized at the C-30 position to glycyrrhetinic acid by CYP72A154 (Seki et al. 2011). Subsequently, two glycosyltransferases, CSyGT and UGT73P12, catalyze sequential glucuronosylations of glycyrrhetinic acid at the C-3 hydroxy group, resulting in the biosynthesis of glycyrrhizin (Nomura et al. 2019, Chung et al. 2020). In the soyasaponin pathway, β-amyrin is oxidized by CYP93E3 to 24-hydroxy-β-amyrin, which is then oxidized to soyasapogenol B by CYP72A566 (Seki et al. 2008, Tamura et al. 2018). Then, CSyGT and some glycosyltransferases add sugars at the C-3 and/or C-22 positions, resulting in the biosynthesis of soyasaponins (Chung et al. 2020). In the oleanolic acid pathway, β-amyrin is oxidized at the C-28 position by CYP716A179 to oleanolic acid (Tamura et al. 2017). In the betulinic acid pathway, 2,3-oxidosqualene is cyclized by LUS1 to lupeol, which is then oxidized by CYP716A179 to betulinic acid (Tamura et al. 2017).

Hairy root culture could be a promising approach for achieving sustainable glycyrrhizin production. Hairy roots are roots induced by infection with *Agrobacterium rhizogenes*. In many cases, hairy roots are capable of producing secondary metabolites that are normally biosynthesized in the roots of differentiated plants, including triterpenoid saponins (Mehrotra et al. 2015, Gutierrez-Valdes et al. 2020, Gantait and Mukherjee 2021). Hairy roots can be cultured easily without phytohormones, grow rapidly and production can be scaled up. It is also possible to produce genetically modified hairy roots by introducing foreign genes into the plant genome via *A. rhizogenes* (Shi et al. 2021). Despite these advantages of hairy root culture, it has rarely been used for glycyrrhizin production due to the extremely low glycyrrhizin content of hairy roots compared to wild licorice roots (Saito et al. 1990). Some reports describe trying to promote glycyrrhizin production in licorice hairy roots. Overexpression of the glycyrrhizin biosynthetic gene and elicitor treatment are representative approaches. Overexpression of CYP88D6 and β-amyrin synthase were attempted separately; the former did not promote glycyrrhizin production (Shirazi et al. 2018), while the latter increased glycyrrhizin accumulation significantly, but the difference from control lines was just 1.6-fold (Wang et al. 2021). Regarding elicitor treatment, methyl jasmonate (100 μM) enhanced glycyrrhizin production in the hairy roots of *G. inflata*, but despite a 5.7-fold increase over the control line, the glycyrrhizin content was as low as 0.1 mg/g DW (Wongwicha et al. 2011). Moreover, determination of the methyl jasmonate concentration and elicitation period is necessary for each strain, in order to achieve substantial production.

Blocking competing pathways in plants has also been tested in attempts to increase production of the target substance. *Panax ginseng* produces two types of tetracyclic triterpenoid saponins, namely protopanaxadiol (PPD)-type saponins and protopanaxatriol (PPT)-type saponins. The accumulation of PPD-type ginsenosides increased in the adventitious roots of *P. ginseng* with disruption of the PPT synthase gene via clustered regularly interspaced short palindromic repeat (CRISPR)/Cas9-based gene editing (Choi et al. 2022). In soybeans, simultaneous disruption of three genes involved in flavonol, anthocyanin and flavone biosynthesis led to an increase in isoflavone content in the hairy roots and plants (Zhang et al. 2020). For licorice, just one example of blocking triterpenoid biosynthesis exists; however, that study targeted the β-amyrin synthase gene for CRISPR/Cas9-based gene editing and did not aim for high glycyrrhizin production (Wang et al. 2021).

In this study, we blocked the pathways that compete with glycyrrhizin by disrupting genes encoding CYP93E3 and CYP72A566 for soyasaponins, CYP716A179 for oleanolic acid and LUS1 for betulinic acid, via CRISPR/Cas9-based gene editing in the hairy roots of *G. uralensis*. We demonstrate that *CYP93E3 CYP72A566* double-knockout and *CYP93E3 CYP72A566 CYP716A179 LUS1* quadruple-knockout hairy roots accumulate glycyrrhizin, accompanied by complete depletion of soyasaponins. Moreover, glycyrrhizin production in hairy roots was further enhanced through the combination of knocking out genes in competing pathways and *CYP88D6*-overexpression. Our findings provide a foundation for sustainable glycyrrhizin production using short-term hairy root culture and a simple purification method that is independent of wild plants.

## Results

### Generation of genome-edited hairy roots

**Fig. 2:**
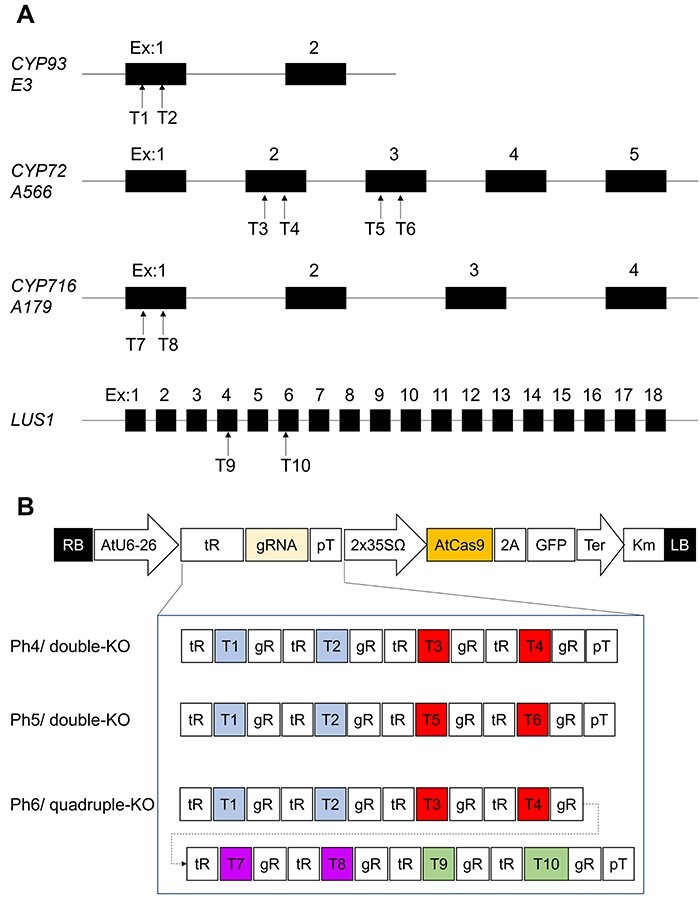
Construction of gRNA and CRISPR/Cas9 binary vector. (A) The exon–intron structure of target genes (not to scale). gRNA target sites (T1–10) are schematically indicated by arrows. Abbreviation: Ex, exon. (B) Schematic representation of CRISPR/Cas9 binary vector in this study. T-DNA cassette of pMgP237-2A-GFP and the composition of the gRNA expression cassette of the CRISPR/Cas9 binary vectors used in this study (Ph4–6) are shown. Plasmids Ph4 and 5 are vectors for constructing double-KO and Ph6 for quadruple-KO. gRNA target sites (T1–10) are color-coded for each target gene. Abbreviations: RB, right border of T-DNA; AtU6-26, *Arabidopsis thaliana* U6 snRNA-26 (U6-26) promoter; tR, tRNA scaffold; gR, gRNA scaffold; pT, poly-T terminator; 2x35SΩ, 2 × *CaMV35S* promoter with the omega enhancer sequence; AtCas9, Arabidopsis-codon optimized *Streptococcus pyogenes* Cas9; 2A, 2 A self-cleavage peptide from *Thosea asigna*; Km, the kanamycin-resistant marker expression cassette; LB, left border of T-DNA.

In our preliminary study, we attempted to promote the production of glycyrrhizin by single knockout of *CYP93E3*, but could not confirm glycyrrhizin production in *CYP93E3*-knockout (KO) hairy roots. In *CYP93E3*-KO hairy roots, soyasapogenol B, an aglycone of soyasaponins, was not detected from GC-MS analysis of the sugar-hydrolyzed metabolites. Instead, sophoradiol, a C-22 oxidative product of β-amyrin, was detected (**Fig. 1**, **Supplementary Fig. S1**). Thus, we decided to generate two types of mutant hairy roots. One, *CYP93E3 CYP72A566* double-knockout (double-KO) hairy roots, leads to a blockage of soyasaponin biosynthesis; the other, *CYP93E3 CYP72A566 CYP716A179 LUS1* quadruple-knockout (quadruple-KO) hairy roots, blocks not only soyasaponin but also oleanolic acid and betulinic acid biosynthesis (**Fig. 1**). The genome sequences of the four target genes were analyzed and *CYP93E3, CYP72A566, CYP716A179*, and *LUS1* gene contained 2, 5, 4 and 18 exons, respectively (**Fig. 2A**). Candidate gRNA target sequences were identified using a web tool and two target sequences were then selected for each gene by their specificity for the target genes and proximity to the 5ʹ-end of the open-reading frame (**Fig. 2A**, **Supplementary Table S1**). Regarding *CYP72A566*, we chose four sequences because two gRNAs from exon-1 failed to lead to Cas9 cleavage in our preliminary examination. A vector system that co-expresses multiplex gRNAs and Cas9 was used (Hashimoto et al. 2018, Nakayasu et al. 2018), and two gRNAs for each gene were inserted into the vector to introduce mutations. To generate double-KO and quadruple-KO, two types of vectors, and one type of vector were constructed, respectively (**Fig. 2B**). The genome-edited hairy roots were then induced from licorice hypocotyls via *A. rhizogenes* infection.

### Screening of *CYP93E3 CYP72A566* double-KO hairy roots

**Fig. 3:**
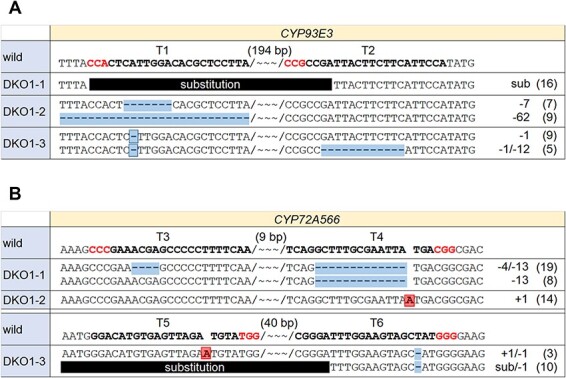
Mutation analysis of the target genes of *CYP93E3 CYP72A566* double-knockout lines. Mutations in the gRNA target regions of *CYP93E3* (A) and *CYP72A566* (B) of double-KO lines are shown. gRNA target and PAM sequences are indicated by bold text. Insertions and deletions are highlighted. Wavy lines signify gaps in the number of base pairs shown above, between the two target sites. The number of PCR amplicons is shown on the right. Abbreviation: sub, substitution.

The screening of double-KO lines was performed by mutation detection followed by sequence analysis in the target sequences. For mutation detection, a microchip electrophoresis device for nucleic acids was used for the analysis of PCR-amplified products of the target regions. When indels caused by CRISPR/Cas9 are small (<4 bp), we could miss the occurrence of mutations. To avoid this, we picked out the lines where changes in DNA size were clearly observed in at least one target gene. Ninety lines were examined and about 40 lines remained as double-KO candidates. Subsequently, sequence analysis revealed that three lines (DKO1-1, 2, 3) had loss-of-function mutations in both *CYP93E3* and *CYP72A566*, due to small deletions and/or insertion or substitution mutations (**Fig. 3**). DKO1-1 and −2 were obtained using the construct Ph4 (**Fig. 2B**), and DKO1-3 was obtained using the construct Ph5 (**Fig. 2B**). Regarding the *CYP93E3* gene in DKO1-1, the region between the two target sites (224 bp) was replaced by 184-bp DNA fragments, and this substitution induced a premature termination codon (**Fig. 3**). In addition, for the *CYP72A566* gene in DKO1-3, two types of mutation were noted: (i) a 1-bp insertion in T5 and a 1-bp deletion in T6, (ii) substitution in T5 and part of T6, with a 1-bp deletion in T6. The former mutation induced a premature termination codon even though a frameshift does not occur. In the latter mutation, the region between the two target sites (77 bp) was replaced by 15-bp DNA fragments and this mutation also induced a premature termination codon.

### Metabolite analysis of *CYP93E3 CYP72A566* double-KO hairy roots

**Fig. 4:**
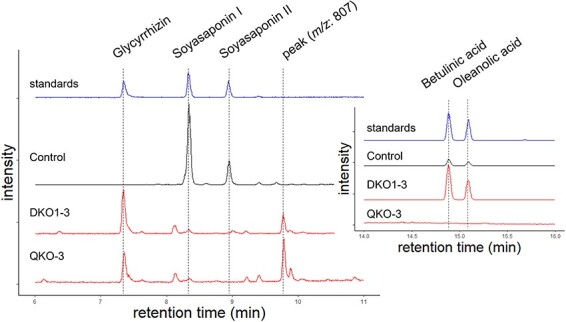
Metabolite analysis of genome-edited hairy roots. LC-MS chromatograms obtained by selected-ion monitoring (SIM) of the theoretical *m/z* values of target compounds. Chromatograms of representative double-KO, quadruple-KO and a control line.

We obtained three double-KO lines by screening hairy roots. Methanol extracts of double-KO lines were then analyzed by LC-MS in SIM mode, looking for the presence of representative triterpenoids including glycyrrhizin. In a control line, soyasaponin I and II were detected as major metabolites among the triterpenoids that we tried to detect, and glycyrrhizin was not detected (**Fig. 4**, **Table 1**). However, in all double-KO lines, soyasaponin I and II were not detected and glycyrrhizin peaks were observed. These double-KO lines contained 333 µg/g DW of glycyrrhizin on an average (**Table 1**). Additionally, the accumulation of an unknown compound with *m/z* 807 eluting at around 9.8 min was confirmed from the chromatogram of double-KO lines. This compound was thought to be 11-deoxo-glycyrrhizin by the value of *m/z*. Furthermore, there was as a more than 10-fold accumulation of oleanolic acid and betulinic acid, on an average, compared to a control line. The content of β-amyrin and soyasapogenol B were then analyzed by GC-MS, using the trimethylsilyl derivatives of the hydrolyzates of the extracts from double-KO lines. We found that more β-amyrin accumulated in the double-KO lines compared to a control line (**Table 1**). Soyasapogenol B, sophoradiol and 24-hydroxy-β-amyrin, which are non-glycosylated intermediates of soyasaponins, were not detected in double-KO lines (**Supplementary Fig. S2**), suggesting that CYP93E3 and CYP72A566 were indeed disrupted. Because a separate experiment showed that the amount of glycyrrhizin secreted into the medium was only 8–17% of the glycyrrhizin accumulated in the hairy roots (**Supplementary Table S2**), more detailed analyses were conducted only on metabolites accumulated in the hairy roots.

**Table 1:** Triterpenoid profiles of *CYP93E3 CYP72A566* double-KO and *CYP93E3 CYP72A566 CYP716A179 LUS1* quadruple-KO lines

	Metabolite content (upper row: µg/g DW, lower row: nmol/g DW)					
Line	β-Amyrin	Glycyrrhizin	Soya saponin I	Soya saponin II	Betulinic acid	Oleanolic acid
Empty vector control	45 (105)	–	2,358 ± 72 (2,500 ± 76)	883 ± 15 (967 ± 16)	59 ± 9 (129 ± 20)	43 ± 6 (94 ± 13)
DKO1-1	(no data)	192 ± 3 (233 ± 4)	–	–	746 ± 14 (1,633 ± 31)	413 ± 7 (904 ± 15)
DKO1-2	211 (494)	250 ± 4 (304 ± 5)	–	–	1,230 ± 23 (2,693 ± 50)	962 ± 30 (2,106 ± 66)
DKO1-3	656 (1,537)	513 ± 11 (623 ± 13)	–	–	441 ± 4 (966 ± 9)	321 ± 2 (703 ± 4)
QKO-1	1,175 (2,754)	530 ± 9 (644 ± 11)	–	–	–	–
QKO-2	(no data)	134 ± 2 (163 ± 2)	–	–	–	–
QKO-3	1,050 (2,461)	274 ± 4 (332 ± 5)	–	–	–	–

β-amyrin was quantified by GC-MS and the other triterpenoids by LC-MS. Minus signs indicate ‘not detected’.

### Screening of *CYP93E3 CYP72A566 CYP716A179 LUS1* quadruple-KO hairy roots

Increased accumulation of oleanolic acid and betulinic acid in *CYP93E3 CYP72A566* double-KO lines motivated us to generate *CYP93E3 CYP72A566 CYP716A179 LUS1* quadruple-KO hairy roots to further improve glycyrrhizin content.

**Fig. 5:**
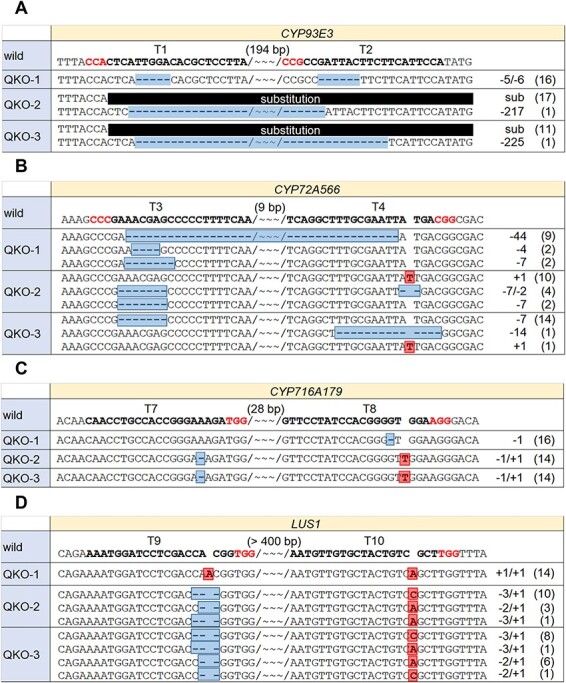
Mutation analysis of the target genes of *CYP93E3 CYP72A566 CYP716A179 LUS1* quadruple-knockout lines. Mutations in the gRNA target regions of *CYP93E3* (A), *CYP72A566* (B), *CYP716A179* (C) and *LUS1* (D) of quadruple-KO lines are shown. gRNA target and PAM sequences are indicated by bold text. Insertions and deletions are highlighted. Wavy lines signify gaps in the number of base pairs shown above, between the two target sites. The number of PCR amplicons is shown on the right. Abbreviation: sub, substitution.

However, screening by mutation detection and sequence analysis becomes labor-intensive as the number of target genes increases because a considerable amount of sequence analysis is required. Therefore, we adopted ELISA-based screening to narrow down quadruple-KO hairy roots. Competitive ELISA was performed using anti-glycyrrhizin monoclonal antibody and candidate quadruple-KO lines were narrowed down by calculating the glycyrrhizin content in the hairy root lysates. Incidentally, the selectivity of the antibody for glycyrrhizin is more than 1,000, three times greater than those against soyasaponin I and glycyrrhetinic acid, respectively. Among the 35 lines, 10 lines were identified with glycyrrhizin content in the lysates that was over 5 µg/g FW, using competitive ELISA. Sequence analysis revealed three lines (QKO-1,2,3) with mutations in all four target genes (**Fig. 5**). Regarding the *CYP93E3* gene in QKO-2 and 3, the region covering part of T1 and all of T2 (313 bp) was replaced by 18-bp DNA fragments, and this substitution induced a premature termination codon. For the *CYP72A566* gene in QKO-2, 7-bp and 2-bp deletions were found in T3 and T4, respectively. These deletions result in an amino acid substitution of 13 residues and amino acid deletion of three residues, suggesting that the correct folding of the CYP72A566 protein is impaired by this mutation. Regarding the *CYP716A179* gene, in QKO-2 and QKO-3, a 1-bp deletion in T7 and a 1-bp insertion in T8 were found; this mutation induces a premature termination codon even though a frameshift does not occur.

### Metabolite analysis of *CYP93E3 CYP72A566 CYP716A179 LUS1* quadruple-KO hairy roots

We obtained three quadruple-KO lines by screening hairy roots. As we expected, betulinic acid and oleanolic acid, as well as soyasaponins, were not detected in the quadruple-KO lines by LC-MS analysis (**Fig. 4**, **Table 1**). Precursors of soyasaponins (sophoradiol, soyasapogenol B and 24-hydroxy-β-amyrin) and betulinic acid (betulin and lupeol) were also not detected by GC-MS analysis (**Supplementary Fig. S3**), suggesting that *LUS1, CYP716A179, CYP93E3* and *CYP72A566* were indeed disrupted by CRISPR-Cas9-mediated gene editing.

However, improvement of glycyrrhizin content was not observed in quadruple-KO lines compared with double-KO lines. The average glycyrrhizin content of the quadruple-KO lines was similar to that of double-KO lines (quadruple-KO: 313 µg/g DW, double-KO: 333 µg/g DW, **Fig. 4**). Instead, the peaks of an unknown compound with m/z 807 eluting around 9.8 min were observed as main saponin in QKO-3 (**Fig. 4**). Moreover, β-amyrin content tended to be higher than that of double-KO (**Table 1**).

### Identification of byproduct of *CYP93E3 CYP72A566* double-KO and *CYP93E3 CYP72A566 CYP716A179 LUS1* quadruple-KO hairy roots

To validate whether the byproduct with *m/z* 807 eluting around 9.8 min is 11-deoxo-glycyrrhizin, we took advantage of an engineered *Saccharomyces cerevisiae* that selectively produces 11-deoxo-glycyrrhizin. The retention time and mass spectrum of the peak of *m/z*:807 were compared between yeast-derived 11-deoxo-glycyrrhizin and extracts of genome-edited hairy roots. We have successfully produced glycyrrhizin in an engineered yeast by introducing seven pathway enzyme genes, including β-amyrin synthase, CYPs and glycosyltransferases, from endogenous 2,3-oxidosqualene (Chung et al. 2020). Excluding only *CYP88D6*, encoding β-amyrin 11-oxidase, from introduced genes generated a yeast strain that selectively produces 11-deoxo glycyrrhizin.

**Fig. 6:**
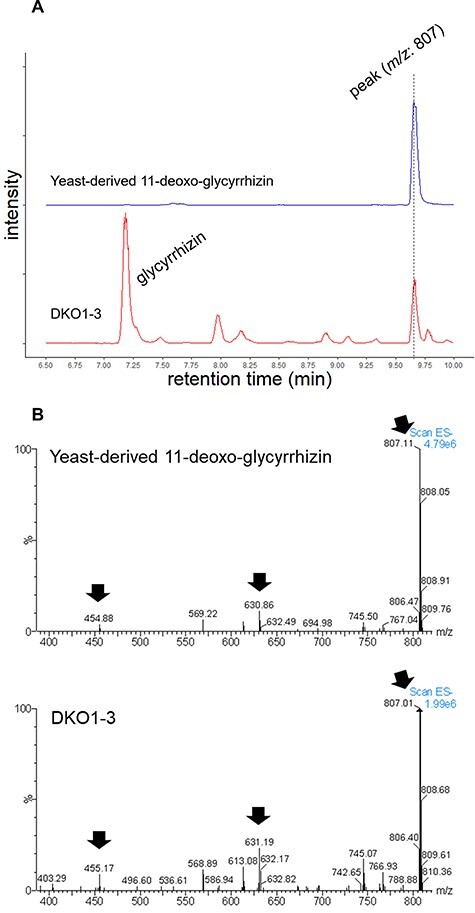
LC-MS analysis of byproduct in *CYP93E3 CYP72A566* double-knockout line. (A) LC-MS chromatograms of yeast-derived 11-deoxo-glycyrrhizin (above) and representative double-KO line (below). Chromatograms were selected based on theoretical *m/z* values of 11-deoxo-glycyrrhizin (807.4) and glycyrrhizin (821.4). (B) Mass spectra of the peaks shown in (A) at a retention time of around 9.65 min. Black arrows represent the corresponding theoretical values for 11-deoxo-glycyrrhizin (807.4), 11-deoxo-glycyrrhetinic acid monoglucuronide (631.4), and 11-deoxo-glycyrrhetinic acid (455.4).

LC-MS analysis in SIM mode showed that the retention time of the peak of *m/z*:807 was almost the same—at around 9.65 min—between yeast-derived 11-deoxo-glycyrrhizin and extracts from the double-KO line (**Fig. 6A**). The mass spectrum from the peaks of *m/z*:807 corresponded as well (**Fig. 6B**). The mass fragments of *m/z*:631, 455 correspond to the values expected with removal of one and two glucuronic acids from 11-deoxo-glycyrrhizin, respectively, suggesting that the mass difference between glycyrrhizin and the byproduct comes from the aglycone part. These data are consistent with our speculation that the byproduct is 11-deoxo-glycyrrhizin.

### Generation of *CYP93E3 CYP72A566* double-KO/*CYP88D6*-OX hairy roots

Accumulation of a substantial amount of 11-deoxo-glycyrrhizin in double-KO lines motivated us to generate hairy roots that combine *CYP93E3 CYP72A566* double-KO and *CYP88D6*-overexpression, henceforth abbreviated to double-KO/*CYP88D6*-OX lines.

**Fig. 7:**
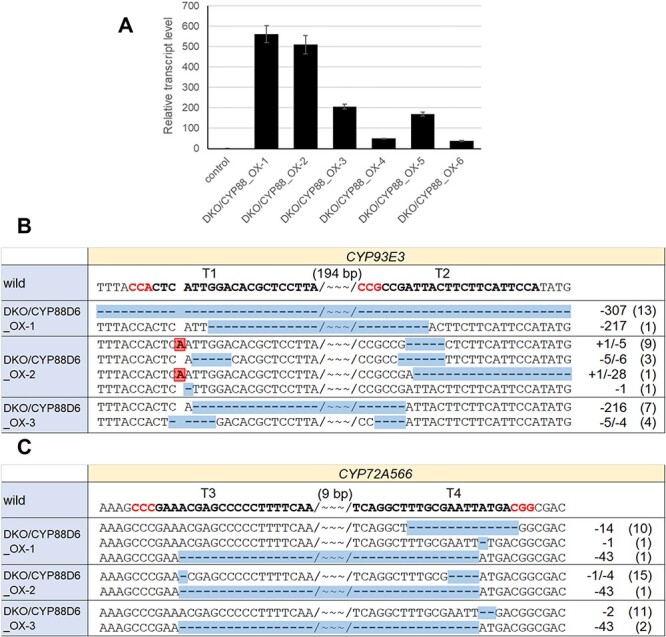
Screening of *CYP93E3 CYP72A566* double-KO/*CYP88D6*-overexpression lines. (A) qPCR analysis o *CYP88D6* gene in double-KO/*CYP88D6*-OX hairy lines. Error bars indicate the SE from three technical replicates. (B) Mutation analysis in the gRNA target regions of *CYP93E3*. (C) Mutation analysis in the gRNA target regions of *CYP72A566*. gRNA target and PAM sequences are indicated by bold text. Insertions and deletions are highlighted. Wavy lines indicate gaps in the number of base pairs shown above, between the two target sites. The number of PCR amplicons is shown on the right.

A *CYP88D6* expression cassette was introduced into the CRISPR/Cas9 vector used for generating double-KO (Ph4) (**Supplementary Fig. S3**), and hairy roots were induced in the same way as before. The double-KO/*CYP88D6*-OX lines were then narrowed down by competitive ELISA, qPCR and sequence analysis. Based on the ELISA results, among 61 hairy roots, six lines were chosen that contained more than 100 µg/g FW of glycyrrhizin and grew well, followed by checking the *CYP88D6* expression level using qPCR analysis. All six lines showed a significantly greater transcription level of *CYP88D6* compared to a control line (**Fig. 7A**). Among these, we selected the three lines (DKO/CYP88_OX-1,2,3) with the greatest transcription level and analyzed the DNA sequence in the target genes, *CYP93E3* and *CYP72A566*. Sequence analysis indeed showed that all three lines had mutations in both target genes (**Fig. 7B, C**). Regarding DKO/CYP88_OX-2, in the *CYP93E3* gene, a 1-bp insertion in T1 and 28-bp deletions in T2 were found and this mutation induced a premature termination codon even though a frameshift did not occur. However, as for DKO/CYP88-OX-3, in the *CYP93E3* gene, (i) 5-bp and 4-bp deletions in T1 and T2, respectively, and (ii) a 216-bp deletion between T1 and T2, were found. The former mutation introduced a premature termination codon, and the latter mutation caused a 72 amino acid deletion. The function of the CYP93E3 protein appears to be affected by this large deletion. As a side note, the strain (GLY-URA-002) used for generation of double-KO/*CYP88D6*-OX hairy roots was different from that of the double-KO and quadruple-KO because the seed stock ran out. Therefore, we again constructed a double-KO using the strain GLY-URA-002, so as to compare the glycyrrhizin content appropriately. Moreover, we generated *CYP88D6*-overexpression (*CYP88D6*-OX) hairy roots, abbreviated as CYP88_OX-1,2 and 3, without knockout mutations to evaluate the effect of the overexpression of *CYP88D6* itself on glycyrrhizin production. The information concerning *CYP88D6*-OX lines and newly constructed double-KO lines (DKO2-1,2,3) such as *CYP88D6* transcription level and mutations in the target genes, is included in the **Supplementary Figs. S4–S6**.

### Metabolite analysis of *CYP93E3 CYP72A566* double-KO/*CYP88D6*-OX hairy roots

**Fig. 8:**
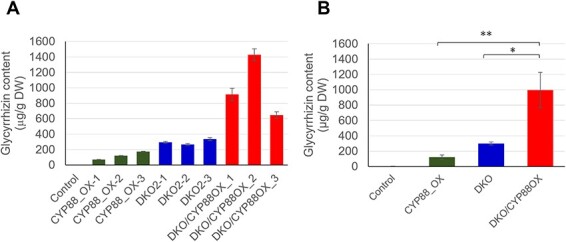
Glycyrrhizin content in engineered hairy roots. (A) Glycyrrhizin content of independent lines of *CYP88D6*-OX, double-KO, and double-KO/*CYP88D6*-OX. Error bars indicate the SE from three technical replicates. (B) The average glycyrrhizin content of three independent lines. Significant differences among the three groups (CYP88_OX, DKO and DKO/CYP88OX) were evaluated using the Tukey-Kramer test. Error bars indicate the SE of three lines. *, ** indicate *P* < 0.05 and *P* < 0.01, respectively.

We selected three lines as double-KO/*CYP88D6*-OX hairy roots and glycyrrhizin content was measured in the lines by LC-MS. As a result of LC-MS analysis, double-KO/*CYP88D6*-OX lines contained about three times more glycyrrhizin than double-KO lines, on average (double-KO/*CYP88D6*-OX: 997 µg/g DW, double-KO: 300 µg/g DW) (**Fig. 8A, B**, **Table 2**). The glycyrrhizin content of *CYP88D6*-OX lines (average: 122.2 µg/g DW) was about two-fifths lower than that of double-KO lines, although the value was higher than the control line. Regarding triterpenoids other than glycyrrhizin, double-KO/*CYP88D6*-OX had low levels of β-amyrin (< 100 µg/g DW) compared with double-KO lines (**Table 2**). The betulinic acid and oleanolic acid content of double-KO/*CYP88D6*-OX lines were also lower than that of double-KO lines. *CYP88D6*-OX lines contained much lower amounts of soyasaponin I and soyasaponin II than the control line. Our study revealed that the effect of *CYP88D6*-overexpression itself on glycyrrhizin production is small; however, the combination of *CYP93E3 CYP72A566* double-KO and *CYP88D6*-overexpression significantly enhanced glycyrrhizin production.

**Table 2:** Metabolic profile of triterpenoids in engineered hairy root lines

	Metabolite content (upper row: µg/g DW, lower row: nmol/g DW)						
Line	β-amyrin	glycyrrhizin	soya saponin I	soya saponin II	betulinic acid	oleanolic acid	GA mono glucuronide
Empty vector control	229 (537)	2 ± 0 (2 ± 0)	948 ± 22 (1,005 ± 23)	698 ± 18 (764 ± 20)	138 ± 2 (302 ± 4)	64 ± 1 (140 ± 2)	–
CYP88_OX-1	(no data)	71 ± 3 (86 ± 4)	30 ± 1 (32 ± 1)	42 ± 0 (46 ± 0)	102 ± 1 (223 ± 2)	11 ± 0 (24 ± 0)	–
CYP88_OX-2	10 (23)	122 ± 1 (148 ± 1)	9 ± 0 (10 ± 0)	16 ± 0 (18 ± 0)	52 ± 1 (114 ± 2)	–	–
CYP88_OX-3	20 (47)	174 ± 6 (211 ± 7)	60 ± 1 (64 ± 1)	95 ± 1 (104 ± 1)	300 ± 5 (657 ± 11)	9 ± 0 (20 ± 0)	–
DKO2-1	630 (1,476)	296 ± 13 (360 ± 16)	–	–	245 ± 53 (536 ± 116)	297 ± 53 (650 ± 116)	–
DKO2-2	668 (1,565)	268 ± 13 (326 ± 16)	–	–	238 ± 44 (521 ± 96)	186 ± 34 (407 ± 74)	–
DKO2-3	99 (232)	299 ± 5 (363 ± 6)	–	–	1,067 ± 14 (2,336 ± 31)	879 ± 1 (1,925 ± 2)	–
DKO/CYP88_OX-1	69 (162)	914 ± 81 (1,111 ± 98)	–	–	–	113 ± 18 (247 ± 39)	62 ± 10 (96 ± 15)
DKO/CYP88_OX-2	73 (171)	1,430 ± 76 (1,738 ± 92)	–	–	116 ± 1 (254 ± 2)	217 ± 2 (475 ± 4)	16 ± 0 (25 ± 0)
DKO/CYP88_OX-3	69 (162)	648 ± 42 (787 ± 51)	–	–	78 ± 18 (171 ± 39)	88 ± 16 (193 ± 35)	26 ± 7 (40 ± 11)

β-Amyrin was quantified by GC-MS and the other triterpenoids by LC-MS. Minus signs indicate ‘not detected’. Abbreviation: GA monoglucuronide, glycyrrhetinic acid monoglucuronide.

## Discussion

Our objective is to achieve high-volume production of glycyrrhizin using hairy root cultures. To accomplish this goal, we disrupted some triterpenoid biosynthesis genes in competing pathways using CRISPR/Cas9-based genome editing to concentrate the metabolic flux into the glycyrrhizin pathway.

The first stage of our investigation evaluated the effect of knockout of two soyasaponin biosynthetic genes (*CYP93E3*/*CYP72A566*) on glycyrrhizin production because soyasaponins are the major triterpenoids in licorice hairy roots and metabolic flux is considered to be largely distributed toward the soyasaponin pathway. The results demonstrate that double-KO hairy roots produce glycyrrhizin in significant amounts, while no glycyrrhizin is detected in the control lines. The average glycyrrhizin content of the double-KO lines was more than twice that of CYP88D6-overexpressing lines, demonstrating a noteworthy effect of blocking the soyasaponin pathway. Based on these results, we speculated that blocking the oleanolic acid and betulinic acid pathways in addition to the soyasaponin pathway would further concentrate the metabolic flux and promote glycyrrhizin production. We thus examined the effect of simultaneous knockout of four triterpenoid biosynthetic genes (*CYP93E3, CYP72A566, CYP716A179, LUS1*); however, an increase in glycyrrhizin production was not confirmed in quadruple-KO lines compared with double-KO lines. In terms of ease of glycyrrhizin purification, quadruple-KO hairy roots are considered superior to double-KO hairy roots because glycyrrhizin and β-amyrin, which are the primary triterpenoids of quadruple-KO (**Table 1**), can be separated by differences in water solubility (Rodrigues et al. 2014, Dai et al. 2018).

We thus suspected the occurrence of branching somewhere in the triterpenoid pathways. The byproduct that newly accumulated in both double-KO and quadruple-KO lines was then analyzed by LC-MS and identified as 11-deoxo-glycyrrhizin, a compound that is not oxidized at the C-11 position, unlike glycyrrhizin. The emergence of this substance suggests that the glycyrrhizin pathway branched off at the point of β-amyrin oxidation, due to the concentration of metabolic flux resulting from disruption of the target genes. Seki et al. showed *in vitro* that CYP88D6, which acts as an oxidase at the C-11 position, takes β-amyrin as a substrate, and not 11-deoxo-glycyrrhetinic acid (Seki et al. 2008). Thus, if the C-30 position of β-amyrin is carboxylated first, oxidation at the C-11 position does not proceed, that is, glycyrrhizin cannot be produced. For this reason, promoting the C-11 oxidation of β-amyrin is presumed to be the key to enhancing glycyrrhizin production; therefore, in the final stage of our investigation, we examined the effect on glycyrrhizin production of the combination of the double-KO and *CYP88D6*-overexpression. As a result, our double-KO/*CYP88D6*-OX line accumulated about 1.4 mg/g DW of glycyrrhizin at best. This content in double-KO/*CYP88D6*-OX lines is on average over three times greater than that in double-KO lines. This increase can be attributed to the synergy between double-knockout and *CYP88D6*-overexpression because *CYP88D6*-OX lines without the double-knockout mutation contained less glycyrrhizin than double-KO lines. The cause of this relatively low glycyrrhizin content of *CYP88D6*-OX lines is unclear. Competition with the intact soyasaponin pathway may have occurred, but the content of soyasaponin I and soyasaponin II in the *CYP88D6*-OX lines was greatly reduced compared to the control line (**Table 2**). Given the low total triterpenoid content of *CYP88D6*-OX lines, metabolites not targeted for detection may have accumulated somewhere. Metabolite analysis with a wider scope of coverage seems required.

To summarize our study, blocking competing pathways in licorice hairy roots by a gene knockout strategy is effective at enhancing glycyrrhizin production. However, production is limited due to the clogging and branching of metabolic flux in the glycyrrhizin pathway. *CYP88D6*-overexpression alone, with no introduction of a gene knockout, is also effective in promoting glycyrrhizin production, but the glycyrrhizin content is the lowest among the hairy roots we generated. On the other hand, the combination of simultaneously blocking the competing pathway and overexpressing the rate-limiting enzyme had a significant effect on promoting glycyrrhizin production. Our results indicate that promoting rate-limiting enzyme expression in the context of the gathering metabolic flux into the glycyrrhizin pathway is important for high glycyrrhizin production. To our knowledge, this is the first report where simultaneous gene knockout and overexpression have been introduced in hairy roots. Genetic engineering of hairy roots has become widely applicable to various plant species, including medicinal plants (Gutierrez-Valdes et al. 2020, Shi et al. 2021). Moreover, instances involving generation of genome-edited hairy roots using the CRISPR/Cas9 system are also increasing (Kiryushkin et al. 2021). We believe that hairy root culture using a combination of overexpression and gene disruption is a promising approach for high-volume production of valuable root secondary metabolites.

As well as glycyrrhizin, 11-deoxo-glycyrrhizin and its corresponding hydrolyzate, 11-deoxo-glycyrrhetinic acid, have been reported to exhibit diverse pharmacological effects (Matsui et al. 2004, Wang et al. 2012, Lin et al. 2014). Glycyrrhizin overdose runs the risk of pseudoaldosteronism due to inhibition of 11-β-hydroxysteroid dehydrogenase 2 (Omar et al. 2012) whereas 11-deoxo-glycyrrhetinic acid inhibits this enzyme less (Shimoyama et al. 2003). Therefore, 11-deoxo-glycyrrhizin is a promising substance with reduced side effects. However, licorice roots contain little 11-deoxo-glycyrrhizin: 0.004% (Kitagawa et al. 1993). In contrast, double-KO and quadruple-KO hairy roots contain 11-deoxo-glycyrrhizin with a peak intensity similar to that of glycyrrhizin; therefore, both types of hairy root could also be used for 11-deoxo-glycyrrhizin production. The accumulation of 11-deoxo-glycyrrhizin resulted from unexpected branching of existing pathways due to the concentration of metabolic fluxes. Our results indicate that hairy roots metabolically engineered by genome editing or overexpression can be used to produce both natural and artificial substances that are rare and valuable.

In this study, we demonstrate that blocking competing pathways in licorice hairy roots enhances glycyrrhizin production. Moreover, the combination of blocking the soyasaponin pathway and *CYP88D6*-overexpression further promotes glycyrrhizin production. Although production is not impressive (∼1.5 mg/g DW) compared to wild plants (20–80 mg/g DW), the culture period for hairy roots is much shorter (1 month), whereas wild licorice takes 3–4 years before it can be harvested. Our results lay the foundation for sustainable glycyrrhizin production. Optimization of culture conditions and elicitor treatments may improve the yield. Wongwicha et al. achieved a 5.7-fold increase in production with methyl jasmonate treatment (Wongwicha et al. 2011). Moreover, the introduction of *CYP72A154*-overexpression in addition to *CYP93E3 CYP72A566* double-KO and *CYP88D6*-overexpression is considered to promote further glycyrrhizin production because we confirmed the accumulation of 11-oxo-β-amyrin, a substrate of CYP72A154, in double-KO/*CYP88D6*-OX hairy roots. Further metabolic modification will lead to improved glycyrrhizin yield in hairy roots.

## Materials and Methods

### Plant materials

The seeds of *G. uralensis* strain GLY-URA-001 and GLY-URA-002 were provided by Health Sciences University of Hokkaido. GLY-URA-001 was used for generating double-KO and quadruple-KO; and GLY-URA-002 for double-KO/*CYP88D6*-OX, *CYP88D6*-OX and double-KO.

### Chemicals

Authentic standards for β-Amyrin, lupeol, oleanolic acid, glycyrrhetinic acid (18β-glycyrrhetinic acid), betulin (Extrasynthese), soyasapogenol B (Tokiwa Phytochemical Co., Ltd., Chiba, Japan), soyasaponin I (Bb), soyasaponin II (Bc) (ChromaDex, Inc., Irvine, CA, USA), betulinic acid, gibberellin A3 (Tokyo Chemical Industry Co., Ltd., Tokyo, Japan), glycyrrhetinic acid-3-O-monoglucuronide (Nacalai Tesque, Inc., Kyoto, Japan) and glycyrrhizin (FUJIFILM Wako Chemical Corporation, Osaka, Japan) were purchased from each reagent company. The 24-hydroxy-β-amyrin was a kind gift from Dr. Kiyoshi Ohyama (Tokyo Institute of Technology).

### Vector construction for hairy root induction

The full lengths of *CYP93E3, CYP72A566, CYP716A179* and *LUS1* were amplified by polymerase chain reaction (PCR) using PrimeSTAR Max DNA Polymerase (Takara Bio Inc., Shiga, Japan) from the extracted genomic DNA of *G. uralensis* (strain GLY-URA-001, Health Sciences University of Hokkaido), and cloned into pJET1.2/blunt Cloning Vector (Thermo Fisher Scientific Inc, Waltham, MA, USA). From randomly selected colonies, the genomic DNA sequences of four target genes were determined. A total of 10 gRNA target sequences (T1-T2 for *CYP93E3*, T3-T6 for *CYP72A566*, T7-T8 for *CYP716A179* and T9–T10 for *LUS1*) were selected using a web tool CRISPRdirect (https://crispr.dbcls.jp/) by applying the DNA sequence of each target gene (**Fig. 2A** and **Supplementary Table S1**).

The primers and plasmids (Ph1-10) used in this study are listed in **Supplementary Tables S3 and S4**. We used the CRISPR/Cas9 binary vector pMgP237-2A-GFP (Ph1), which is capable of co-expressing multiple gRNAs and the CRISPR/Cas9 protein (Hashimoto et al. 2018, Nakayasu et al. 2018). Binary vector plasmids for *Agrobacterium* transformation were constructed by following the method of Nakayasu et al. To increase the efficiency of transcription from the U6 promoter, a single G was added to the 5ʹ-end of the T1–T4, T6, T7, T9 and T10. The three types of DNA fragments with (i) T1 and T2, (ii) T2 and T3, and (iii) T3 and T4, at their 5ʹ- and 3ʹ-ends were amplified by PCR from plasmid Ph2 using primers P01/02, P03/04 and P05/06, respectively. Plasmid Ph4 was then constructed by introducing these PCR fragments into the *Bsa*I site of plasmid Ph1 using Golden Gate cloning methods (**Fig. 2**) (Engler et al. 2008). In the same way, plasmid Ph5 was constructed from the DNA fragments with (I) T1 and T2, (II) T2 and T5 and (III) T5 and T6 at both ends using primers P01/02, P03/07, and P08/09, respectively. Plasmid Ph6 was constructed from the DNA fragments with (I) T1 and T2, (II) T2 and T3, (III) T3 and T4, (IV) T4 and T7, (V) T7 and T8, (VI) T8 and T9, (VII) T9 and T10 at both ends using primers P01/02, P03/04, P05/10, P11/12, P13/14, P15/16 and P17/18, respectively. To construct plasmid Ph7 by the In-Fusion^TM^ cloning method, the linear vector DNA fragment and a *CYP88D6* expression cassette consisting of 35S *Cauliflower Mosaic Virus* (CaMV) promoter, *A. thaliana ADH* 5ʹ-UTR, *CYP88D6* and *HSP* terminator were amplified by PCR from the plasmid Ph4 and Ph3 using primers P31/32 and P33/34, respectively. The vector and insert fragments were mixed with In-Fusion HD Enzyme (Takara Bio Inc) to obtain plasmid Ph7 (**Supplementary Fig. S3**). Plasmid Ph10 was generated by transferring a *CYP88D6* expression cassette consisting of *Lotus japonicus ubiquitin* promoter, *CYP88D6* and *HSP* terminator, from plasmid Ph 9 to plasmid Ph8 using a GATEWAY LR reaction. *Agrobacterium rhizogenes* ATCC15834 was transformed by electroporation using the plasmids Ph1, Ph4-7 and Ph10.

### Induction of licorice hairy roots and hairy root culture

The hairy root lines generated in this study are listed in **Supplementary Table S5**. The hairy root lines of DKO1a, DKO1b, QKO and control (empty vector) were induced with *Agrobacterium* harboring the binary vectors of Ph1, Ph4, Ph5 and Ph6, respectively, using the hypocotyls of *G. uralensis* strain GLY-URA-001. The hairy root lines of DKO2, DKO/CYP88_OX, CYP88_OX and control (empty vector) were induced by *Agrobacterium* harboring the binary vectors of Ph1, Ph4, Ph7 and Ph10, respectively, using the hypocotyls of the *G. uralensis* strain GLY-URA-002. We induced hairy roots according to Tamura and colleagues (Tamura et al. 2018) with slight modifications. The co-culture period was 3 days. After co-culture, the seedlings were transferred into 1/2 MS medium supplemented with 1% sucrose, 125 µg/mL cefotaxime and 0.2% gellan gum every 2 weeks before the isolation of hairy roots. Six weeks after infection, hairy roots were isolated from seedlings and placed on 1/2 McCown woody plant medium (Duchefa Biochemie, Haarlem, Netherlands) supplemented with 1% sucrose, 0.01 µM gibberellin A3, 125 µg/mL cefotaxime and 0.8% agar, pH 5.8 (1/2WP plate) and cultured for approximately 1 month. The temperature and light conditions were maintained at 22°C with a 16 h light/8 h dark cycle from germination to plate culture after isolation. Hairy roots cultured on 1/2 WP plates were harvested, freeze-dried, homogenized and used for genotyping. When genetic mosaicism was found, hairy roots were cultured for 2–4 months, with monthly subcultures used for re-genotyping. For metabolite analysis, hairy roots cultured on 1/2 WP plates were transferred into 100 mL of 1/2 McCown woody plant medium supplemented with 1% sucrose and 125 µg/mL cefotaxime (pH 5.8), and cultured in the dark for 30 days with shaking (90 rpm at 25°C).

### Heteroduplex mobility assay

The hairy roots were removed from the hypocotyls and about 1 cm of severed root was homogenized with BioMasher II (Nippi. Inc, Tokyo, Japan) in buffer solution (100 mM Tris–HCl, pH 9.5, 1 M KCl, 10 mM EDTA). After incubation at 95°C for 10 min, the supernatant (hereinafter referred to as hairy root lysate) was used as a PCR template. The regions surrounding the gRNA target sites were amplified using the relevant primers (P19/20 for *CYP93E3*, P21/22 for *CYP72A566*-T3/T4, P23/24 for *CYP72A566*-T5/T6, P25/26 for *CYP716A179*, and P27/28 for *LUS1*) and KOD FX Neo DNA polymerase (Toyobo Co., Ltd.). The PCR products were analyzed on a micro-tip electrophoresis machine (MultiNA; Shimadzu Corporation).

### Competitive ELISA assay

Competitive ELISA assay was performed according to the method of Shan et al. (Shan et al. 2001). Anti-glycyrrhizin antibodies and glycyrrhizin-BSA were provided by Sumitomo Chemical Co., Ltd. The changes to their methodology are noted as follows. Fifty microliters of glycyrrhizin-BSA (in PBS) was immobilized on the immune plates. For blocking, 200 µL of Blocking One (Nacalai Tesque, Inc.) was used. The addition of antibodies was divided into two steps: (I) 50 µL of anti-glycyrrhizin (0.01 µg/mL), and (II) 100 µL of anti-mouse IgG (0.25 µg/mL, Jackson ImmunoResearch Inc, West Grove, PA, USA). When changing the antibodies, the plate was washed three times with 200 µL of TPBS. For detection, ELISA POD Substrate TMB Solution Easy (Nacalai Tesque, Inc.) was used and the absorbance was measured at 450 nm using a microplate reader SPARK (Tecan Trading AG).

### Sequence analysis

The region containing the two gRNA target sequences of each target gene was amplified by PCR using the same primer sets used in the HMA, and PCR products were cloned into the pJET1.2/blunt Cloning Vector. Colony PCR was performed from randomly selected colonies using primers P29/30 and KOD FX Neo DNA polymerase. After cleaning up using a Gel/PCR Extraction Kit (Fast Gene), PCR products were mixed with ExoSAP-IT (Thermo Fisher Scientific Inc.) at 37°C for 30 min. Solutions diluted in pure water to appropriate concentrations were used for sequence analysis.

### qPCR

Total RNA was extracted using a Plant Total RNA Extraction Mini Kit (FAVORGEN Biotech Corporation, Ping Tung, Taiwan) from hairy roots grown for 1–1.5 months on a culture plate after isolation. The RNA obtained was purified using the After Tri-Reagent RNA Clean-Up Kit (FAVORGEN Biotech Corporation) after digesting contaminated genomic DNA with recombinant DNase I (RNase-free) (Takara Bio Inc.). First-strand cDNA was synthesized from purified total RNA by PrimeScript RT Master Mix (Perfect Real Time) (Takara Bio Inc.). We performed qPCR analysis with Light Cycler 96 (F. Hoffmann-La Roche, Ltd, Basel, Switzerland) and FastStart Essential DNA Green Master (F. Hoffmann-La Roche, Ltd.) using primers P35–38 (**Supplementary Table S3**). The expression of the β-tubulin gene was analyzed as a reference gene following Seki et al. (Seki et al. 2008).

### Generation of 11-deoxo-glycyrrhizin producing yeast and extraction of yeast-derived 11-deoxo-glycyrrhizin

Yeast generation was performed according to the method of glycyrrhizin-producing yeast (Chung et al. 2020). The yeast strains and plasmids used in this study are listed in **Supplementary Tables S6** and **S7**. The yeast strain 11d-GA, which is engineered to produce 11-deoxo-glycyrrhetinic acid, was transformed using plasmid Py1 to produce yeast strain 11d-GAM. A second transformation was then performed, using plasmid Py2 and strain 11d-GAM to produce yeast strain 11d-GC. Yeast-derived 11-deoxo-glycyrrhizin was extracted from this strain 11d-GC. The extraction of the product from yeast followed the method of Chung et al. (2020).

### Metabolites extraction from licorice hairy roots for LC-MS analysis

Dried hairy roots (5 mg) were extracted with methanol. The samples were vortexed for 30 s and sonicated for 20 min. After centrifugation, the supernatants were collected. The collection was repeated a total of three times. The collected solution was evaporated and resuspended in 500 µL of methanol.

### LC-MS analysis

LC-MS analysis was performed according to Chung et al (Chung et al. 2020) with slight modifications. The mobile phase was composed of 0.5% (v/v) acetic acid in water (solvent 1) and 0.5% (v/v) acetic acid in acetonitrile (solvent 2). Samples were separated via gradient elution with 30% solvent 2 for 2 min to 100% over 13 min (45% at 8 min and 100% at 15 min) at a flow rate of 0.30 mL/min. The final condition was maintained for 4.0 min and returned to the initial condition, resulting in a total chromatography run time of 33.0 min. The values used for SIM for each metabolite analysis are listed in **Supplementary Table S8**.

### Metabolite extraction from licorice hairy roots for GC-MS

Dried hairy roots (10 mg) were mixed with 10 µL of internal standard (uvaol, 100 ppm) and extracted with methanol. The samples were vortexed and sonicated for 60 min. After the liquid had evaporated, 1 mL methanol and 1 mL 4 M HCl were added to the precipitates and the mixture hydrolyzed at 80°C for 1 h. The hydrolyzed products were extracted with hexane/ethyl acetate (1:1, v/v) and evaporated. The precipitates were resuspended in 500 µL of methanol. Subsequently, 100 µL of solution was evaporated and derivatized with 100 μL N-methyl-N-(trimethylsilyl) trifluoroacetamide (Sigma-Aldrich) at 80°C for 30 min before GC-MS analysis.

### GC-MS analysis

GC–MS analysis was performed according to Romsuk et al (Romsuk et al. 2022).

## Supplementary Material

pcad161_Supp

## Data Availability

All data underlying this article are available in the article and the online supplementary data.
