# Supplementary material for: Glycyrrhizin Production in Licorice Hairy Roots Based on Metabolic Redirection of Triterpenoid Biosynthetic Pathway by Genome Editing
Source: Plant Cell Physiol. 2023 Dec 28;65(2):185–98. doi: 10.1093/pcp/pcad161 (PMC10873519; doi:10.1093/pcp/pcad161)
Supplement: pcad161_Supp [file pcad161_supp.zip › suppl_data/pcp-2023-e-00205-File010.pdf]

Supplementary Tables

Supplementary Table S1 gRNA target sequences. Bold letters indicate PAM sequences.

Target gene	Name	target sequences (5' to 3')
CYP93E3	T1	TAAGGAGCGTGTCCAATGAG**TGG**
	T2	TGGAATGAAGAAGTAATCGG**CGG**
CYP72A566	T3	TTGAAAAGGGGGCTCGTTTC**GGG**
	T4	TCAGGCTTTGCGAATTATGA**CGG**
	T5	GGACATGTGAGTTAGATGTA**TGG**
	T6	CGGGATTTGGAAGTAGCTAT**GGG**
CYP716A179	T7	CAACCTGCCACCGGGAAAGA**TGG**
	T8	GTTCCTATCCACGGGGTGGA**AGG**
LUS1	T9	AAATGGATCCTCGACCACGG**TGG**
	T10	AATGTTGTGCTACTGTCGCT**TGG**

Supplementary Table S2 Comparison of glycyrrhizin levels in hairy roots and culture medium

Line	culture period (week)	glycyrrhizin content in hairy roots (μg/g DW)	dry weight of hairy roots (mg)	glycyrrhizin ammount in hairy roots (μg)	glycyrrhizin concentration in medium (nM)	medium volume after harvest (mL)	glycyrrhizin ammount in medium (μg)	Percentage of glycyrrhizin amount in hairy roots (%)
A	4	280 ± 13	214	60 ± 3	159 ± 11	94	12 ± 1	83
A	8	189 ± 13	553	105 ± 7	165 ± 5	84	11 ± 0	90
B	3	185 ± 13	409	76 ± 6	83 ± 7	92	6 ± 1	92

Double-KO lines were cultured in 100 mL of 1/2 McCown woody plant medium supplemented with 1% sucrose and 125 μg/mL cefotaxime (pH 5.8) for the periods listed in the table above with shaking at 90 rpm at 25°C in the dark. Glycyrrhizin content in hairy roots and culture medium was measured using competitive ELISA. Dried hairy roots (1 mg) were suspended in buffer solution (100 mM Tris-HCl, pH 9.5, 1 M KCl, 10 mM EDTA). After incubation at 95°C for 10 min, the supernatant was diluted 200 and 400 times with water and used as the hairy root fraction. The culture medium was filtered using Sartolab RF (250 mL/ PS/ 0.22μm) (Sartorius AG) and diluted 3, 9, 27, and 81 times with water and used as the medium fraction. The amount of glycyrrhizin was calculated by selecting results for dilution factors that fell within the concentration range of the calibration curves created using the authentic standard. Glycyrrhizin levels in the hairy roots and medium were evaluated by culturing two lines for different durations, and results showed that more than 80% glycyrrhizin was present in the hairy roots under all conditions.

Supplementary Table S3 Primers used in this study. Small letters indicate *Bsa*I adapters. Underlined sections represent 15 bp of overhang for infusion cloning.

No.	sequence (5' to 3')	Comment
P01	ttgggtctcgTGCAGTAAGGAGCGTGTCCAATGAGGTTTTAGAGCTAGAAATAGCA	construction of pMgP237-doubleKO_1, doubleKO_2, quadrupleKO
P02	ttgggtctccTTCTTCATTCCACTGCACCAGCCGGGAATCGAA	construction of pMgP237-2A-GFP [double-KO; T1/T2/T3/T4], pMgP237-2A-GFP [double-KO; T1/T2/T5/T6], pMgP237-2A-GFP [quadruple-KO; T1/T2/T3/T4/T7/T8/T9/T10]
P03	ttgggtctcgAGAAGTAATCGGGTTTTAGAGCTAGAAATAGCA	construction of pMgP237-2A-GFP [double-KO; T1/T2/T3/T4], pMgP237-2A-GFP [double-KO; T1/T2/T5/T6], pMgP237-2A-GFP [quadruple-KO; T1/T2/T3/T4/T7/T8/T9/T10]
P04	ttgggtctccCCCCCTTTTCAACTGCACCAGCCGGGAATCGAA	construction of pMgP237-2A-GFP [double-KO; T1/T2/T3/T4], pMgP237-2A-GFP [quadruple-KO; T1/T2/T3/T4/T7/T8/T9/T10]
P05	ttgggtctcgGGGGCTCGTTTCGTTTTAGAGCTAGAAATAGCA	construction of pMgP237-2A-GFP [double-KO; T1/T2/T3/T4], pMgP237-2A-GFP [quadruple-KO; T1/T2/T3/T4/T7/T8/T9/T10]
P06	ttgggtctccAAACTCATAATTCGCAAAGCCTGACTGCACCAGCCGGGAATCGAA	pMgP237-2A-GFP [double-KO; T1/T2/T3/T4]
P07	ttgggtctccACTCACATGTCCTGCACCAGCCGGGAATCGAA	pMgP237-2A-GFP [double-KO; T1/T2/T5/T6]
P08	ttgggtctcgGAGTTAGATGTAGTTTTAGAGCTAGAAATAGCA	pMgP237-2A-GFP [double-KO; T1/T2/T5/T6]
P09	ttgggtctccAAACATAGCTACTTCCAAATCCCGCTGCACCAGCCGGGAATCGAA	pMgP237-2A-GFP [double-KO; T1/T2/T5/T6]
P10	ttgggtctccCGCAAAGCCTGACTGCACCAGCCGGGAATCGAA	pMgP237-2A-GFP [quadruple-KO; T1/T2/T3/T4/T7/T8/T9/T10]
P11	ttgggtctcgTGCGAATTATGAGTTTTAGAGCTAGAAATAGCA	pMgP237-2A-GFP [quadruple-KO; T1/T2/T3/T4/T7/T8/T9/T10]
P12	ttgggtctccGAGGATCCATTTCTGCACCAGCCGGGAATCGAA	pMgP237-2A-GFP [quadruple-KO; T1/T2/T3/T4/T7/T8/T9/T10]
P13	ttgggtctcgCCTCGACCACGGGTTTTAGAGCTAGAAATAGCA	pMgP237-2A-GFP [quadruple-KO; T1/T2/T3/T4/T7/T8/T9/T10]
P14	ttgggtctccTAGCACAACATTCTGCACCAGCCGGGAATCGAA	pMgP237-2A-GFP [quadruple-KO; T1/T2/T3/T4/T7/T8/T9/T10]
P15	ttgggtctccGGTGGCAGGTTGCTGCACCAGCCGGGAATCGAA	pMgP237-2A-GFP [quadruple-KO; T1/T2/T3/T4/T7/T8/T9/T10]
P16	ttgggtctcgGCTACTGTCGCTGTTTTAGAGCTAGAAATAGCA	pMgP237-2A-GFP [quadruple-KO; T1/T2/T3/T4/T7/T8/T9/T10]
P17	ttgggtctcgCACCGGGAAAGAGTTTTAGAGCTAGAAATAGCA	pMgP237-2A-GFP [quadruple-KO; T1/T2/T3/T4/T7/T8/T9/T10]
P18	ttgggtctccAAACTCCACCCCGTGGATAGGAACTGCACCAGCCGGGAATCGAA	pMgP237-2A-GFP [quadruple-KO; T1/T2/T3/T4/T7/T8/T9/T10]
P19	CACCATGCTTGACATCCAAGGCTACCTCGT	HMA for *CYP93E3*
P20	TCCCGGTCTTCGATATCTCCAGAACATTC	HMA for *CYP93E3*
P21	GGAAGAATTCATTTATGTGGCTTGGTCCG	HMA for *CYP72A566*-T3/T4
P22	CTTCATTTTCTCTACATTGAATGCTGGGC	HMA for *CYP72A566*-T3/T4
P23	GGCAACATTGCTTCTAGTATTTACATATAGTTTG	HMA for *CYP72A566*-T5/T6
P24	GAAGTACCAAACTTCCCCACTTTTTAG	HMA for *CYP72A566*-T5/T6
P25	CACCATGGAGCATTTCTACATGTCCCTTCTC	HMA for *CYP716A179*
P26	GGTAGACGGTGACATGCGTCTTTTCCTC	HMA for *CYP716A179*
P27	GAGGGTCATAGCACAATGTTTGGATC	HMA for *LUS1*
P28	CCAGTTAATTTGATTATAAGGCTC	HMA for *LUS1*
P29	CGACTCACTATAGGGAGAGCGGC	Sequence for the flanking region of target sites
P30	AAGAACATCGATTTTCCATGGCAG	Sequence for the flanking region of target sites

No.	sequence (5' to 3')	Comment
P31	TTAACACGTCTTCTCGAATTAGTTTCTCC	construction of pMgP237-2A-GFP [double-KO; T1/T2/T3/T4, 35S/*CYP88D6*/HSPter]
P32	TACGTAGTCTCACGTTACAATTAGTAC	construction of pMgP237-2A-GFP [double-KO; T1/T2/T3/T4, 35S/*CYP88D6*/HSPter]
P33	ACGTGAGACTACGTAAAGCTTGCATGCCTGCAGGTC	construction of pMgP237-2A-GFP [double-KO; T1/T2/T3/T4, 35S/*CYP88D6*/HSPter]
P34	GAGAAGACGTGTTAAGCCGCAGGTACCAATTCCTTATC	construction of pMgP237-2A-GFP [double-KO; T1/T2/T3/T4, 35S/*CYP88D6*/HSPter]
P35	TCTTCGCAAACTGGCAGTGA	qPCR for *β-tublin*
P36	CGAGATGTGAGTGGGGCAAA	qPCR for *β-tublin*
P37	ATGGACGAAAATTGGAGGACGA	qPCR for *CYP88D6*
P38	CTGGTTGCTGTACTTTCATGGC	qPCR for *CYP88D6*

Supplementary Table S4   Plasmids used in this study.

No.	plasmid	Backbone
Ph1	pMgP237-2A-GFP	Nakayasu et al.
Ph2	pMD_gtRNA	Nakayasu et al.
Ph3	pRI 201-AN DNA [p35S-CYP88D6-HSPter]	pRI 201-AN DNA (Takara bio)
Ph4	pMgP237-2A-GFP [double-KO; T1/T2/T3/T4]	Ph1
Ph5	pMgP237-2A-GFP [double-KO; T1/T2/T5/T6]	Ph1
Ph6	pMgP237-2A-GFP [quadruple-KO; T1/T2/T3/T4/T7/T8/T9/T10]	Ph1
Ph7	pMgP237-2A-GFP [double-KO; T1/T2/T3/T4, p35S-CYP88D6-HSPter]	Ph1
Ph8	pSD8 [pUB-GW-HSPter]	Suzuki et al.
Ph9	pENTR/DTOPO [CYP88D6]	pENTR/D-TOPO (Thermo Fisher Scientific)
Ph10	pSD8 [pUB-CYP88D6-HSPter]	Ph8

Suzuki, H., Takahashi, H., Fukushima, E. O., Nakazono, M., Muranaka, T., Seki, H. (2022). Identification of basic helix-loop-helix transcription factors that activate betulinic acid biosynthesis by RNA-sequencing of hydroponically cultured Lotus japonicus. *bioRxiv*. DOI:10.1101/2022.11.16.516519

Supplementary Table S5 Hairy root lines generated in this study.

Hairy root lines	Background	Plasmid used for construction, Mutation type
Control (empty vector)	GLY-URA-001	Ph1, control
DKO1a (2 lines)	GLY-URA-001	Ph4, double-KO
DKO1b (1 line)	GLY-URA-001	Ph5, double-KO
QKO (3 lines)	GLY-URA-001	Ph6, quadruple-KO
Control (empty vector)	GLY-URA-002	Ph1, control
DKO2 (3 lines)	GLY-URA-002	Ph4, double-KO
DKO/CYP88_OX (3 lines)	GLY-URA-002	Ph7, double-KO/*CYP88D6* -OX
CYP88_OX (3 lines)	GLY-URA-002	Ph8, *CYP88D6* -OX

Supplementary Table S6 Plasmids for generation of 11-deoxo-glycyrrhizin-producing yeast.

No.	plasmid	backbone
Py1	pESC-HIS [GAL10/ UGD; GAL1/ *Gu* CSyGT]	Chung et al.
Py2	pESC-URA [GAL10/ CYP72A63; GAL1/ UGT73P12]	Chung et al.

Supplementary Table S7 Yeast strains used in this study.

Strain	Strain; Plasmid used for construction	Source
INVSc1	MATa his3Δ1 leu2 trp1-289 ura3-52 MAT his3Δ1 leu2 trp1-289 ura3-52	Thermo Fisher Scientific
11d-GA	INVSc1; pYES3 [ADH1/ bAS], pESC-LEU [GAL10/ CPR], pYES-DEST52 [GAL1/ CYP72A63]	Chung et al (not published)
11d-GMG	11d-GA; Py1	This study
11d-GC	11d-GMG; Py2	This study

Supplementary Table S8   List of standards used in LC-MS analysis.

No.	Compound	Molecular Formula	*m/z*
1	betulinic acid	$C_{30}H_{48}O_3$	455.4
2	oleanolic acid	$C_{30}H_{48}O_3$	455.4
3	glycyrrhetinic acid	$C_{30}H_{46}O_4$	469.3
4	glycyrrhetinic acid-3-*O* -monoglucuronide	$C_{36}H_{54}O_{10}$	645.4
5	11-oxo-30-hydro-glycyrrhizin	$C_{42}H_{64}O_{14}$	791.4
6	soyasaponin III	$C_{42}H_{68}O_{14}$	795.5
7	11-deoxo-glycyrrhizin	$C_{42}H_{64}O_{15}$	807.4
8	30-hydroxy-glycyrrhizin	$C_{42}H_{64}O_{15}$	807.4
9	glycyrrhizin	$C_{42}H_{62}O_{16}$	821.4
10	soyasaponin II	$C_{47}H_{76}O_{17}$	911.5
11	soyasaponin I	$C_{48}H_{78}O_{18}$	941.5

Supplementary Figures

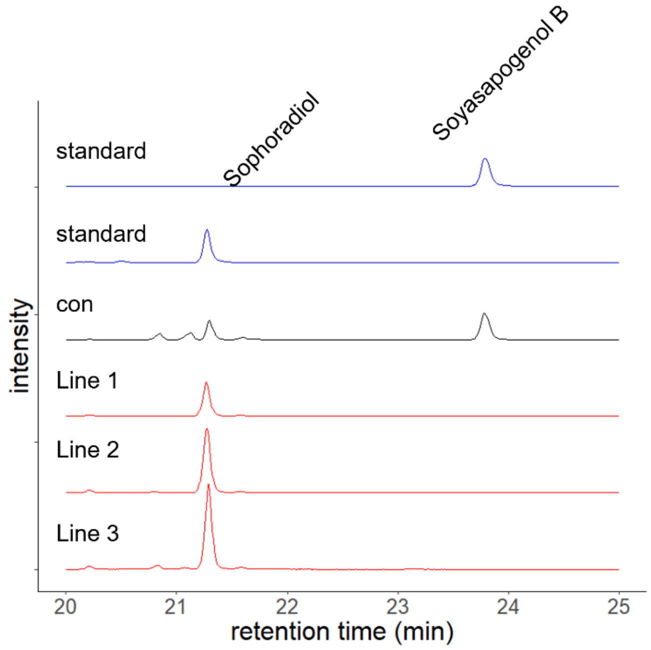

Fig. S1 Metabolite analysis of *CYP93E3*-KO hairy roots. GC-MS analysis of *CYP93E3*-KO lines. Extracted ion chromatograms at *m/z* 306 of three *CYP93E3*-KO lines (Line1-3) and the empty vector control line. Abbreviations: con, control.

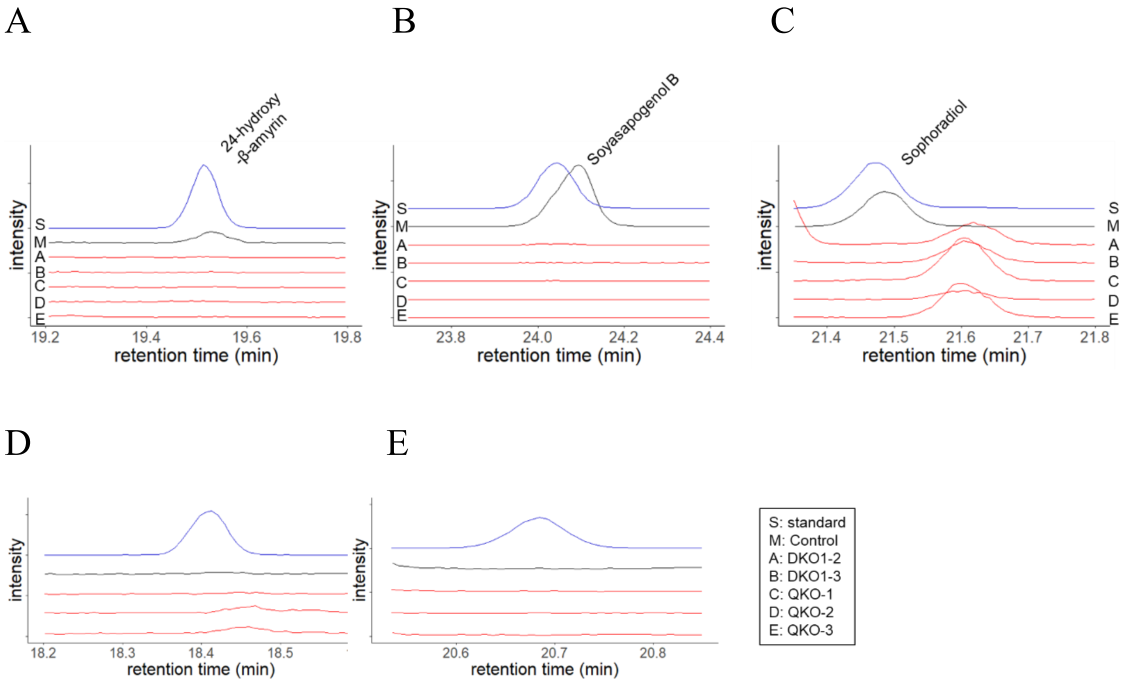

Fig. S2 GC-MS analysis of *CYP93E3 CYP72A566* double-knockout and *CYP93E3 CYP72A566 CYP716A179 LUS1* quadruple-knockout lines. Extracted ion chromatograms showing precursors of triterpenoids at *m/z* 218 (A), 306 (B and C), 189 (D and E) of double-KO and quadruple-KO lines and the empty vector control line.

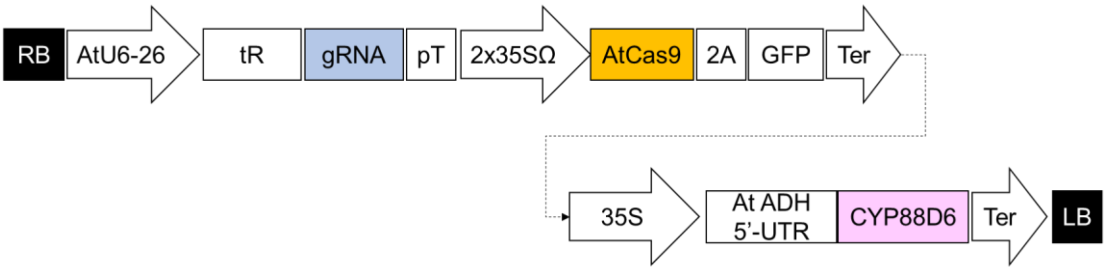

Fig. S3 CRISPR/Cas9 binary vector of *CYP93E3 CYP72A566* double-knockout/*CYP88D6*-overexpression hairy roots. Abbreviations: RB, right border of T-DNA; AtU6-26, *Arabidopsis thaliana* U6 snRNA-26 (U6-26) promoter; tR, tRNA scaffold; gR, gRNA scaffold; pT, poly-T terminator; 2x35SΩ, 2×*CaMV35S* promoter with the omega enhancer sequence; AtCas9, *Arabidopsis*-codon optimized *Streptococcus pyogenes* Cas9; 2A, 2 A self-cleavage peptide from *Thosea asigna*; 35S, *CaMV35S* promoter; At ADH 5'-UTR, *Arabidopsis*-alcohol dehydrogenase 5'-untranslated region; Ter, HSP terminator; LB, left border of T-DNA.

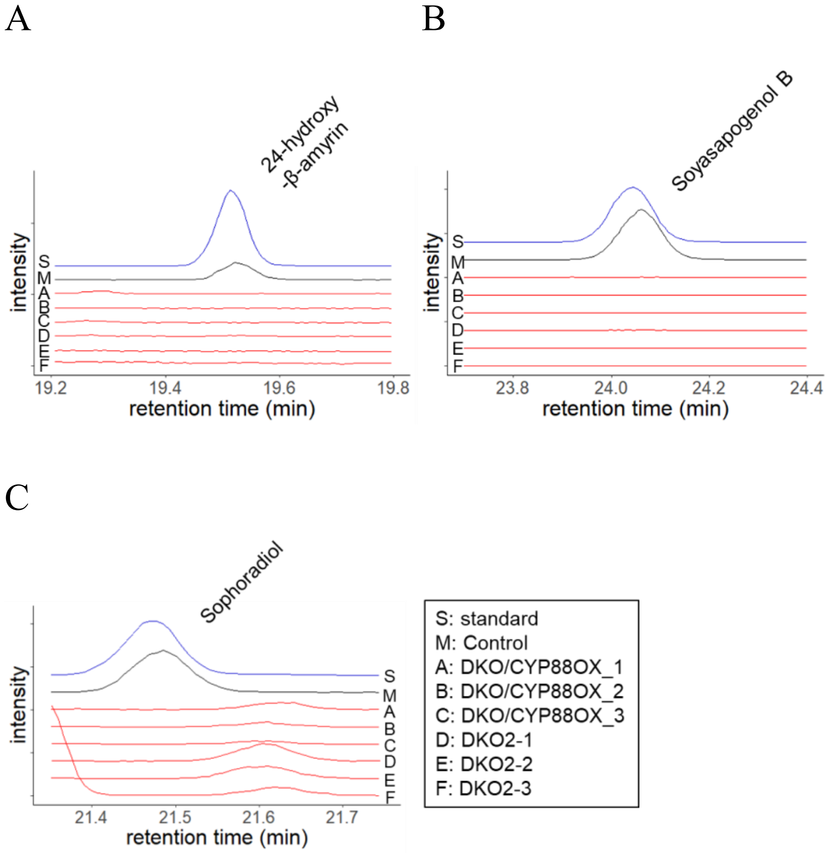

Fig. S4 GC-MS analysis of *CYP93E3 CYP72A566* double-knockout/*CYP88D6*-overexpression lines. Extracted ion chromatograms showing precursors of soyasaponins at *m/z* 218 (A), 306 (B and C) of double-KO/CYP88D6-OX, double-KO and the empty vector control line.

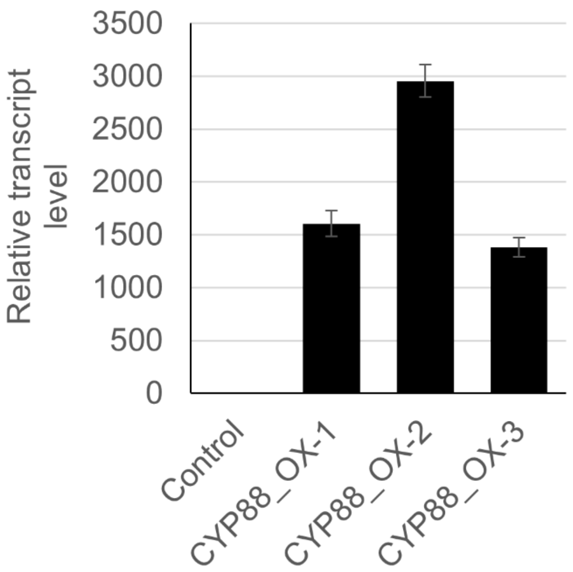

Fig. S5 qPCR analysis of *CYP88D6* gene in *CYP88D6*-OX lines. Error bars indicate the SE from three technical replicates.

A

	CYP93E3		
wild	T1 (194 bp) T2<br>TTTA**CCACTC ATTGGACACGCTCCTTA**/~~~/**CCGCCGATTACTTCTTCATTCCA**TATG		
DKO2-1	TTTACCACTC**A**ATTGGACACGCTCCTTA/~~~/CCGCCGATTACTTCTTCATTCCATATG	+1	(13)
DKO2-2	---------- -----------------/~~~/---------ACTTCTTCATTCCATATG	-258	(11)
	TTTACCACTC ATT--------------/~~~/-------TTACTTCTTCATTCCATATG	-217	(1)
DKO2-3	TTTACCACTC A substitution ATTACTTCTTCATTCCATATG	sub	(11)

B

	CYP72A566		
wild	T3 (9 bp) T4<br>AAAG**CCCGAAACGAGCCCCCTTTTCAA**/~~~/**TCAGGCTTTGCGAATTA TGACGG**CGAC		
DKO2-1	AAAGCCCGAAACGAGCCCCCTTTTCAA/~~~/TCAGGCTTTGCGAATT- -GACGGCGAC	-2	(13)
	AAAGCCCGAA------CCCCTTTTCAA/~~~/TCAGGCTTTGCGAATT- TGACGGCGAC	-6/-1	(2)
DKO2-2	AAAGCCCGAA-----------------/~~~/----------------A TGACGGCGAC	-43	(6)
	AAAGCCCGAAACGAGCCCCCTTTTCAA/~~~/TCAGGCTTTGCG----A TGACGGCGAC	-4	(3)
	AAAGCCCGAAACGAGCCCCCTTTTCAA/~~~/TCAGGCTTTGCGAATTA**G**TGACGGCGAC	+1	(1)
DKO2-3	AAAGCCCGAAACGAGCCCCCTTTTCAA/~~~/TCAGGCTTT-------- -----GCGAC	-13	(14)
	AAAGCCCGAA-----CCCCCTTTTCAA/~~~/TCAGGCTTTGCGA---- ---------C	-5/-13	(1)

Fig. S6 Mutation analysis of the target genes of *CYP93E3 CYP72A566* double-knockout (GLY-URA-002). Mutations in the gRNA target regions of *CYP93E3* (A) and *CYP72A566* (B) of double-KO lines are shown. gRNA target and PAM sequences are indicated by bold black and red. Insertions are highlighted in red. Deletions are represented on a light blue background, with dashes. Substitutions are indicated on a black background. Wavy lines mean gaps in the number of base pairs shown above, between the two target sites. The number of PCR amplicons is shown on the right. Abbreviations: sub, substitution.

Regarding DKO2-2, in the *CYP93E3* gene, one of two types of mutation was a 258 bp deletion and this mutation causes an 86 amino acid deletion. The function of the CYP93E3 protein appears to be affected by this large deletion. Regarding DKO2-3, in the *CYP93E3* gene, the region between the two target sites (216 bp) were replaced by 215 bp of inverted DNA fragments and this substitution induces a premature termination codon. In the *CYP72A566* gene, one of two types of mutations was a 5 bp and 13 bp deletion in T3 and T4, respectively. These deletions cause an amino acid substitution of 11 residues and amino acid deletion of six residues, suggesting that the correct folding of the CYP72A566 protein is impaired by this mutation.
